# Distinct Successions of Common and Rare Bacteria in Soil Under Humic Acid Amendment – A Microcosm Study

**DOI:** 10.3389/fmicb.2019.02271

**Published:** 2019-10-01

**Authors:** Pengfa Li, Jia Liu, Chunyu Jiang, Meng Wu, Ming Liu, Zhongpei Li

**Affiliations:** ^1^State Key Laboratory of Soil and Sustainable Agriculture, Institute of Soil Science, Chinese Academy of Sciences, Nanjing, China; ^2^University of Chinese Academy of Sciences, Beijing, China; ^3^Soil and Fertilizer & Resources and Environment Institute, Jiangxi Academy of Agricultural Sciences, Nanchang, China

**Keywords:** rare bacteria, succession, community assembly, niche breadth, functional diversity, humic acid

## Abstract

Humic acid (HA) is widely used for soil quality improvement, yet little is known how bacterial communities, especially common and rare bacteria, respond to HA amendment, which is crucial to understand biodiversity and function in agroecosystem. Therefore, a manipulated microcosm experiment with a gradient of HA amendment was conducted to unveil this. The results showed that common and rare taxa had similar patterns in species richness, while rare taxa exhibited a higher turnover, which caused their higher structural dissimilarity. Common species with wider niche breadths were more strongly influenced by deterministic filtering when compared to rare taxa, which occupied narrow niches and were primarily controlled by stochastic processes. Generally, species with wider niche breadths were always more strongly influenced by deterministic selection. The analysis of predicted functions revealed that rare taxa occupied more unique predicted functional traits than common taxa, suggesting that rare taxa played a key role in maintaining the functional diversity. In addition, there was a significant positive correlation between species richness and predicted functional diversity in rare taxa rather than common taxa. Our findings highlight the distinct structural and predicted functional successions of common and rare bacteria in soil under HA amendment.

## Introduction

Humic substances are the most widely-spread natural complexing ligands occurring in nature, of which humic acid (HA) is the most explored group of humic substances, and the remarkable properties of HA have attracted the attention of many investigators (Peña-Méndez et al., 2005). Nowadays, HA is widely being used in many areas. As agronomic measures, humic materials are frequently used as additives in fertilizers, and their soil quality regulating effects, such as promoting soil fertility, improving soil moisture and optimizing soil chemical composition and physical texture, have been numerously documented (Khaled and Fawy, 2011; Olaetxea et al., 2018). However, how soil bacterial communities respond to HA amendment, which is crucial to understanding biodiversity and function in agroecosystem, is little known. This calls for further investigation on successions of soil bacterial communities under HA amendment.

A central goal of microbiology is to understand the successions in microbial communities and the corresponding driving factors (Shade et al., 2013). There are two divergent patterns describing the succession in species compositions of bacterial communities, turnover and nestedness (Harrison et al., 1992; Baselga, 2010). Turnover implies the replacement of some species by others, and thus many species will never co-occur together (Ulrich and Gotelli, 2007). In contrast, nestedness refers to the extent to which the species composition of small assemblages is a sub-set of the species composition of larger assemblages (Wright and Reeves, 1992). Existing studies on microbial succession have mainly focused on entire communities (Kazemi et al., 2016; Ranjitkar et al., 2016; Prest et al., 2018), while few have paid attention to rare species. Microorganisms are perhaps the most diverse (Torsvik et al., 2002) and abundant (Whitman et al., 1998) organisms on Earth, and they typically show a skewed species abundance distribution, with relatively few dominant species co-existing alongside a high number of rare species (Nemergut et al., 2011). Moreover, some rare microbial taxa have been shown to provide specific and important functions within their communities, such as establishing a functional cache or resource pool for responding to environmental changes (Jones and Lennon, 2010), maintaining species diversity (Dohrmann et al., 2013), and promoting functional redundancy (Louca et al., 2018). In agroecosystems, soil rare taxa occupy the root system of major crops that may determine plant performance (Hol et al., 2010; Saleem et al., 2016). However, a lack of understanding of the successional patterns of common and rare taxa have largely limited our ability to better predict the variations in structure and ecological functions of bacterial communities.

Deterministic and stochastic processes are two divergent ecological processes that drive succession in bacterial communities (Chase and Myers, 2011; Zhou and Ning, 2017). Deterministic processes (e.g., environmental filters and biotic filters) are directional and converge toward a stable state (Anderson, 2007; Zhou and Ning, 2017). In contrast, stochastic processes (i.e., ecological drift, dispersal, and stochastic diversification) reflect random changes in the relative abundance of species and are not associated with environmentally derived fitness (Chase and Myers, 2011; Zhou and Ning, 2017). Common and rare taxa vary in niche breadth, number of taxonomic groups, and affordability to environmental changes, and these differentiation may result in contrasting ecological assemblages of communities. For instance, the wider niches and higher competitiveness for resources of common taxa will cause them to be more strongly influenced by deterministic processes than rare taxa (Orrock and Watling, 2010; Umana et al., 2015). In addition, rare taxa are vulnerable to ecological drift (Nemergut et al., 2013), which would also significantly drive stochastic assemblages of rare taxa (Zhou and Ning, 2017). However, common taxa may remain stable under given environmental conditions because of their high stability, but such environmental conditions may cause remarkable variations in rare taxa because of their high sensitivity (Clarke and Murphy, 2006; Jiao et al., 2017a). Based on this, assemblages of rare taxa may tend to be more deterministic than those of common taxa. Overall, the ecological assemblages and key processes shaping succession in bacterial communities remain unclear in common and rare taxa under soil environmental changes.

A comprehensive understanding of the successional patterns of common and rare taxa can help us better predict the variations in microbial functions. Microbial communities play key roles in maintaining multiple ecosystem functions and services simultaneously, including nutrient cycling,34 primary production, litter decomposition, and climate regulation (Bardgett and van der Putten, 2014; Wagg et al., 2014; Yan et al., 2017). Microbial biodiversity also plays an important role in maintaining soil health that is an important ecosystem services (Saleem et al., 2019). The common taxa have a greater ability to resist environmental changes; thus, they may play important roles in maintaining the functional stability of the bacterial communities (Jiao et al., 2017b). Furthermore, rare taxa may maintain the functional diversity of the communities because of their high quantity of species (Jiao et al., 2017b). Specially, we want to clarify if the species stability of common taxa can also lead to a functional stability, and if the species sensitivity of rare taxa would result in large functional variation.

In this study, a manipulated microcosm system with different HA additions was applied to investigate the successions of common and rare bacteria using high-throughput sequencing and ecological models. Specifically, the following questions were addressed: (1) Are common and rare taxa following similar successional patterns under HA amendment? (2) What are the main ecological processes shaping succession in common and rare taxa? (3) Is there succession of functions along with succession of species composition? Through bioinformatics analysis, we observed distinct structural and predicted functional successions of common and rare taxa under HA amendment. Our findings provide a better understanding of the distinct structural and predicted functional responses of common and rare taxa in soil to HA amendment.

## Materials and Methods

### Incubation Experiment

Soil samples were collected from the Ecological Experimental Station of Red Soil at the Chinese Academy of Sciences in Yujiang, Jiangxi Province (28°13′ N, 116°55′ E). The experimental soil was classified as Udic Ferrosol (FAO1998 classification), which is commonly known as red soil in China. Sampling was conducted in late October 2017, which was shortly after the peanut harvest. Twenty random surface soil cores (0–20 cm) were collected from each field and used as one composite sample. The fresh soil samples were air-dried and sieved (2 mm) after visible crop roots were removed. The HAs were extracted from a lignite from Shanxi following a modified method based on the procedure developed by the International Humic Substances Society (Wu et al., 2016). The structural features of HA are presented in Supplementary Table S1.

Ninety six 250 ml aseptic plastic bottles were used to conduct the incubation experiment. Each bottle contained an air-dried equivalent of 30 g of soil. Freeze-dried HAs were added into each plastic bottle at concentrations ranging from 0 to 1.8 g (Table 1), after which the bottles were shaken to thoroughly mix the soil and HA. The soil water content was then adjusted to a 60% water holding capacity with deionized water. All pots were covered with sterile membranes that permitted gaseous exchange to maintain aerobic conditions and then incubated in the dark at 25°C. Each treatment had 12 replicates. Throughout the entire course of the incubation, deionized water was added every 7 days to maintain a constant soil moisture content. On days 7, 15, 30, and 60 after incubation, three replicates were randomly and destructively sampled from each treatment. One portion of a fresh soil sample was then air-dried to determine the soil organic carbon (SOC), total nitrogen (TN), available nitrogen (AN), and available phosphorus (AP), while the remainder of the soil samples were stored at −20°C until DNA extraction and analyses. The soil properties are presented in Supplementary Table S2.

**TABLE 1:** Humic acid (HA) addition amount in each treatment.

**Treatment**	**CK**	**H1**	**H2**	**H3**	**H4**	**H5**	**H6**	**H7**
HA addition amount	0	0.15 g	0.30 g	0.60 g	0.90 g	1.20 g	1.50 g	1.50 g

### Soil DNA Extraction, Bacterial 16S rRNA Amplification, Illumina Sequencing, and Processing of Sequencing Data

Soil DNA was extracted from 0.5 g of soil (fresh weight) using a Fast^®^ DNA SPIN Kit (MP Biomedicals, Santa Ana, CA, United States). The extracted DNA was quantified by spectrophotometer and quality-evaluated by gel electrophoresis. The universal bacterial PCR primers 515F (5′-GTGCCAGCMGCCGCGGTAA-3′) and 907R (5′-CCGTCAATTCCTTTGAGTTT-3′) (Biddle et al., 2008) targeting the V4–V5 hypervariable region of the 16S rRNA genes were used to amplify 16S rRNA gene fragments from each sample. PCR reactions were conducted by subjecting the samples to the following conditions: 94°C for 5 min, followed by 35 cycles at 94°C for 30 s, 50°C for 30 s, and 72°C for 30 s. PCR products were then sequenced on the Illumina MiSeq PE250 platform (Illumina, Inc., San Diego, CA, United States). DNA extraction, bacterial 16S rRNA amplification and high-throughput sequencing were done by a biotechnology company (Majorbio Bio-Pharm Technology, Co., Ltd., Shanghai, China).

Raw sequence data were analyzed using the Quantitative Insights into Microbial Ecology (QIIME) pipeline (Caporaso et al., 2010^1^). QIIME quality trimming was performed using the following criteria: (1) sequence lengths should be greater than 200 bp; (2) average quality scores should be greater than 25; (3) no ambiguous bases; (4) sequences should match the primer and barcode. After quality trimming, we obtained 4,491,201 high quality sequences of bacterial 16S rRNA gene (between 31,352 and 71,073 sequences per sample). High-quality sequences were then combined and analyzed using the MOTHUR software package (Schloss et al., 2009). Clustering of sequences into operational taxonomic units (OTUs) at a 97% nucleotide similarity level was performed using UCLUST (Edgar, 2010). All samples were then rarefied to 31,352 sequences per sample to evaluate the beta diversity of the bacterial communities and the most abundant sequence from each OTU was selected as a representative sequence. Subsequently, a representative sequence from each OTU was aligned against the SILVA 132 reference alignment using the RDP classifier (Wang et al., 2007). Tax4Fun^2^ was used to predict the potential functions of the bacterial communities based on the SILVA assignments (Aßhauer et al., 2015). A phylogenetic tree was first constructed using Fasttree after aligning the OTU representative sequences with PyNAST (Caporaso et al., 2010). The 16S rRNA gene sequences were submitted to the NCBI Sequence Read Archive (SRA) under the Accession No. SRP 169560.

### Statistical Analysis

The definition of common and rare taxa was based on the relative frequency of each OTU (Lynch and Neufeld, 2015). Common taxa were those appearing in > 90% of all samples, while rare taxa were those appearing in less than a quarter of all samples. Alpha-diversity indexes were calculated in QIIME using script “alpha_diversity.py.” The phylogenetic diversity (PD) of bacterial communities was calculated based on the phylogenetic tree that constructed following the method mentioned above. Statistically significant differences in species richness among treatments were determined by one-way analysis of variance (ANOVA) tests, along with the use of Duncan’s test for multiple comparisons (*P* < 0.05). To calculate dissimilarities in species or predicted functional compositions among samples, a non-metric multidimensional scaling (NMDS) based Bray–Curtis matrix was generated and a two-way PERMANOVA was carried out using the PAST software (version 3). Niche breadth was calculated according to Levin’s niche breadth index equation (Levins, 1968). The turnover and nestedness of the bacterial communities were calculated using the “betapart” R package based on the presence/absence OTU table (Baselga, 2010). A partial Mantel test was employed to determine the correlation between the species or predicted functional composition and variations in temporal distance, HA application amount and soil properties.

The βNTI (beta nearest taxon index) was determined to display the assembly processes of bacterial communities (Stegen et al., 2012). The phylogenetic tree and OTU table were then used to determine the βMNTD (beta mean nearest taxon distance) and NTI (nearest taxon index). The βMNTD is the mean phylogenetic distance to the closest relative between pairs of communities, and the βNTI is the between-assemblage analog of the NTI. The R package “picante” was used to determine the values. Briefly, βNTI was determined as follows:

$$\beta \,\mathrm{NTI}=\frac{\beta \,{\mathrm{MNTD}}_{\text{obs}}-\bar{\beta \,{\mathrm{MNTD}}_{\text{null}}}}{\mathrm{SD}\,\left({\mathrm{NMTD}}_{\text{null}}\right)}$$

βNTI values < −2 or > + 2 indicate that the observed βMNTD was more than two standard deviations away from the mean of the null βMNTD distribution (which is the stochastic expectation). As such, βNTI values that are < −2 or > + 2 indicate there a statistically significant divergence between the observed and expected βMNTD, which is interpreted as less than or greater than the expected phylogenetic turnover, respectively (Stegen et al., 2012).

A neutral assembly model was used to determine the potential contribution of neutral processes to the bacterial community assembly by predicting the relationship between OTUs occurrence frequency and their relative abundance (Sloan et al., 2006). This model predicts that rare taxa will be lost with time due to ecological drift, while abundant taxa are more likely to be dispersed by chance and thus present in more samples. We further applied the PD theory to confirm the microbial community assembly. We used the edge-length abundance distribution (EAD) method to investigate whether PDs of bacterial communities were more or less than the expected PD of a randomly generated null-modeled local community from a metacommunity (O’Dwyer et al., 2012). Briefly, a PD lower than the expected PD represents that environmental filtering dominates community assembly, whereas a PD higher than the expected PD indicates that community assembly is more governed by competitive exclusion (Mouquet et al., 2012).

Random forest analyses were performed to quantitatively evaluate the important predictors to predicted functional diversity of bacterial communities. The importance of each predictor was determined by assessing the decrease in prediction accuracy [that is, the increase in the mean square error (MSE) between observations and predictions] when the data for the predictor was randomly permuted. This decrease was averaged over all trees to produce the final measure of importance. These analyses were conducted using the ‘*randomForest*’ package of the R statistical software. The significance of predictor importance was assessed by using the ‘*rfPermute*’ package. According to the random forest outputs, structural equation modeling (SEM) has been employed to model complex relationships between directly and indirectly observed (latent) factors (Grace et al., 2012). In our study, SEM analysis was used to gain a mechanistic understanding of how HA and incubation time mediate alterations in predicted functional diversity of bacterial communities. The model was tested by the robust maximum likelihood evaluation method using the Amos 17.0 software package (Smallwaters Corporation, Chicago, IL, United States). The χ^2^ values, degree freedom, and *P*-values, were adopted to evaluate the structural equation model fitness (Hooper et al., 2008). The best fitting and most parsimonious model were obtained after excluding all non-significant parameters.

## Results

### General Patterns of Species Richness

Overall, sequencing of 16S rRNA genes yielded 4,491,201 high-quality sequences and 5,153 OTUs at the 97% similarity level after excluding singletons. At the phylum level, *Chloroflexi* (28.40%), *Acidobacteria* (20.62%), *Actinobacteria* (10.05%), *Alphaproteobacteria* (6.41%), *Betaproteobacteria* (5.89%), and *Firmicutes* (5.84%) dominated all samples (Supplementary Figure S1). For the total dataset, 808 OTUs (15.68%) representing 91.11% of all sequences were highly common, whereas 2,832 OTUs (54.96%) contributing to 1.19% of all sequences were affiliated with rare taxa. The species composition of common taxa was similar to that of all taxa, while *Planctomycetes*, *Gamma-proteobacteria*, and *Delta-proteobacteria* were dominant in rare taxa (Supplementary Figure S1). Generally, among common bacteria, relative abundance of oligotrophic taxa was significantly higher than that of copiotrophic taxa; while among rare bacteria, relative abundance of copiotrophic taxa was significantly higher (Supplementary Table S3). Across all three datasets, HA added amount was always significantly positively correlated with relative abundance of copiotrophic taxa, yet significantly negatively correlated with relative abundance of oligotrophic taxa (Supplementary Table S4).

All, common, and rare taxa showed similar patterns in species richness with increases in the amount of HA added (Supplementary Figure S2). Briefly, species richness peaked in treatment H1, then decreased significantly as HA was added across all four sampling stages. Pearson’s correlation tests showed that HA addition amount and soil properties, including SOC, TN, AN, and AP, were always negatively correlated with species richness. Incubation time was only significantly correlated with species richness of rare taxa, and pH was always positively correlated with species richness (Table 2). A two-way ANOVA confirmed that HA addition had greater effects on species richness when compared to incubation time (Supplementary Table S5). In addition, the absolute values of slopes between environmental factors (except AN) and species richness of rare taxa were always higher than those of common taxa (Table 2). Similar to species richness, other alpha-diversity indexes, including Chao1, ACE, Shannon-Wiener, and PD also peaked in H1 treatment, and HA addition always had greater negative effects on these alpha-diversity indexes (Supplementary Figure S2 and Supplementary Table S5). Additionally, the absolute values of slopes between environmental factors and these other alpha-diversity indexes of rare taxa were also always higher than those of common taxa (Supplementary Table S6).

**TABLE 2:** The top panel shows the correlations between species richness and humic acid (HA) addition amount, incubation time, and soil properties.

	**All taxa**	**Common taxa**	**Rare taxa**
**Pearson’s correlation between richness and**			
HA	–0.852^∗∗∗^	–0.624^∗∗∗^	–0.715^∗∗∗^
Time	–0.048	0.015	−0.230^∗^
pH	0.412^∗∗∗^	0.268^∗∗^	0.370^∗∗∗^
SOC	–0.832^∗∗∗^	–0.592^∗∗∗^	–0.692^∗∗∗^
TN	–0.856^∗∗∗^	–0.613^∗∗∗^	–0.739^∗∗∗^
AN	–0.265^∗∗^	–0.293^∗∗^	–0.133
AP	–0.843^∗∗∗^	–0.598^∗∗∗^	–0.737^∗∗∗^
**Partial Mantel tests of temporal distance against**			
Total β-diversity	0.454^∗∗∗^	0.041	0.353^∗∗∗^
Turnover	0.413^∗∗∗^	0.050	0.367^∗∗∗^
Nestedness	–0.058	–0.008	–0.118
**Partial Mantel tests of difference in HA addition amount against**			
Total β-Diversity	0.448^∗∗∗^	0.138^∗∗^	0.294^∗∗∗^
Turnover	0.195^∗∗∗^	–0.087	0.174^∗∗∗^
Nestedness	0.644^∗∗∗^	0.333^∗∗∗^	0.336^∗∗∗^

*The lower panel shows the correlations between temporal distances, differences in HA addition amount and total beta diversity, turnover, and nestedness. Coefficients were determined by Pearson’s correlation tests and partial Mantel tests, respectively. HA, humic acid addition amount; Time, incubation time; SOC, soil organic carbon; TN, total nitrogen; AN, available nitrogen; AP, available phosphorus; ^∗^, ^∗∗^, and ^∗∗∗^ represent significant correlations at *P* < 0.05, 0.01, and 0.001, respectively.*

### Succession in Species Composition

**FIGURE 1:**
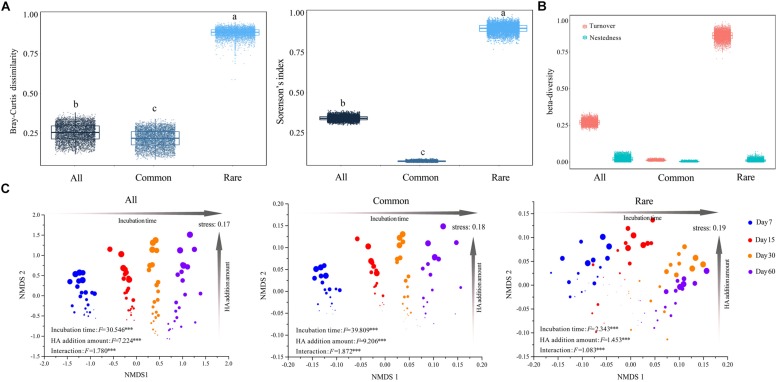
Succession in species composition of bacterial communities that consist of different taxa. **(A)** Mean pairwise Bray–Curtis dissimilarity and sørensen’s index among taxa. The top and bottom boundaries of each box indicate the 75^th^ and 25^th^ quartile values, respectively, and lines within each box represent the median values (*n* = 4560). Different letters above bars indicate significant differences at the *P* < 0.05 level according to non-parametric Mann–Whitney *U-*test. **(B)** Turnover and nestedness in all, common, and rare taxa. All: whole bacterial communities; Common: common bacterial communities; Rare: rare bacterial communities. **(C)** NMDS based on Bray–Curtis distance of whole, common, and rare bacterial communities, respectively. Circles in different color represent samples collected from different stages. Larger sizes indicate more added HA and vice versa. Value *F* and significance for incubation time, HA addition amount, and interaction (the interactive effects of incubation time and HA addition amount) were determined by two-way PERMANOVA. ^∗∗∗^*P* < 0.001.

Both pairwise Bray–Curtis dissimilarity and sørensen’s index among all taxa were significantly greater than that among common taxa, but significantly lower than that among rare taxa (Figure 1A). An NMDS plot based on the Bray–Curtis distance showed that bacterial communities can be clustered into different groups according to either incubation time or HA addition amount, regardless of how many species the community contained (Figure 1C). Two-way PERMANOVA showed that incubation time, rather than HA addition amount, always had greater effects on species composition across all three datasets (Supplementary Table S7). In addition, partial Mantel tests also confirmed that incubation time had a greater influence on species composition when compared to HA addition amount or soil properties including pH, SOC, TN, AN, and AP, except that the correlation between AP and community composition of rare species was slightly stronger than that of incubation time and community composition of rare species (Supplementary Figure S3).

Turnover among all taxa was significantly higher than that among common taxa, whereas it was significantly lower when compared to rare taxa (Figure 1B). Across all three datasets, turnover always played key roles in driving changes of species composition (Figure 1B). Partial Mantel tests showed that, in both whole communities (all taxa) and rare bacterial communities (rare taxa), temporal distance was significantly correlated with turnover while differences in HA addition amount were significantly correlated with both components (turnover and nestedness, partial Mantel *P* < 0.001, Table 2). However, temporal distance were significantly correlated to neither turnover nor nestedness of common taxa (partial Mantel *P* > 0.05, Table 2), whereas HA was significantly correlated with nestedness of common taxa (partial Mantel *P* < 0.001, Table 2).

### Ecological Assemblages of Bacterial Communities

**FIGURE 2:**
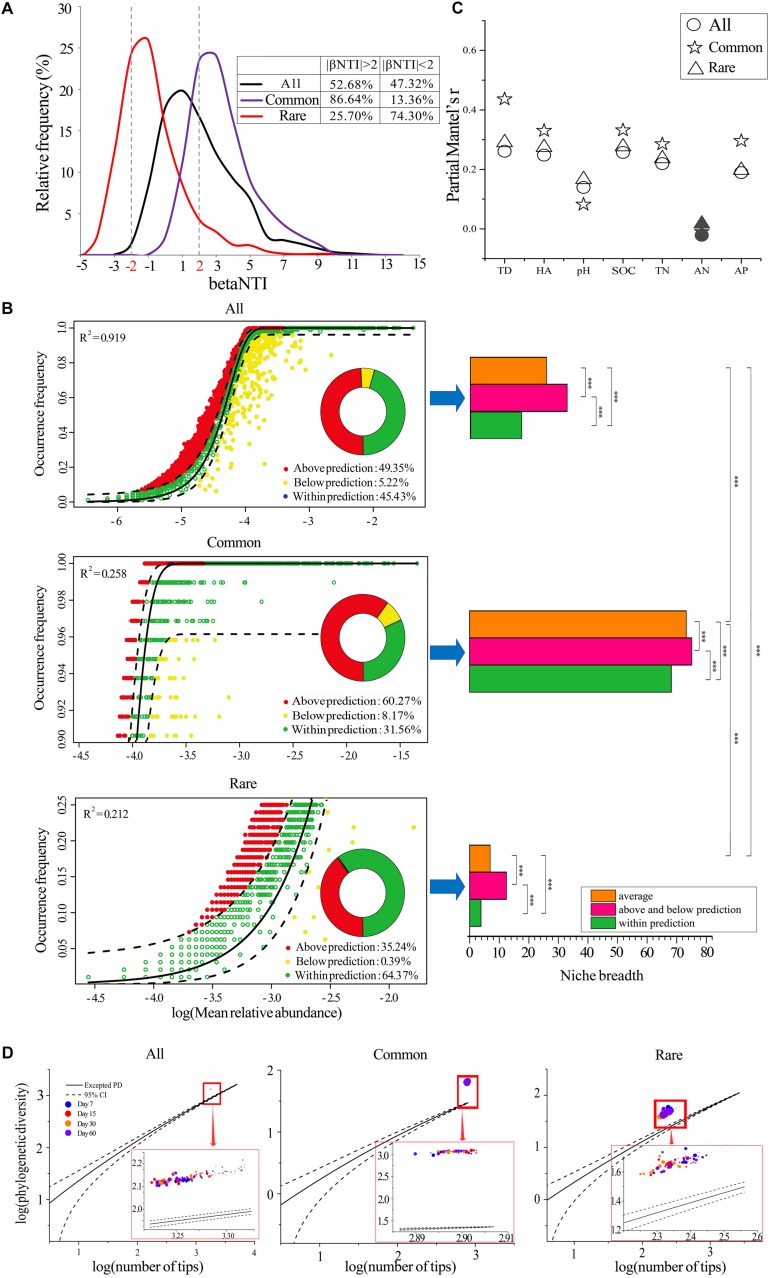
Assemblages of bacterial communities. **(A)** The distributions of community analogs (betaNTI) in different datasets. Left panel represents the fit of the neutral model for all **(B)**, common **(C)**, and rare **(D)** taxa. Right panel represents the niche breadths of species. The predicted occurrence frequency is shown as a solid yellow line, and dashed yellow lines represent 95% confidence intervals around the model prediction. OTUs that occur more or less frequently than predicted by the neutral model are shown in different colors. R2 indicates the fit to the neutral model. ^∗∗∗^, significant difference of niche breadths between different species groups at *P* < 0.001 according to the non-parametric Mann–Whitney *U-*test. All, whole taxa (*n* = 5153); Common, common taxa (*n* = 808); Rare, rare taxa (*n* = 2832). **(C)** Correlations between betaNTI and temporal distance, HA addition amount, and soil properties. Coefficients were determined by Mantel tests. Solid symbols represent a non-significant correlation. TD, temporal distance. HA, difference in humic acid (HA) addition amount. **(D)** Assessment of the community assembly process using EAD. The solid lines represent the expected PDs and dashed lines represent 95% confidence intervals. Circles of different color represent samples collected from different stages. Larger sizes indicate more added HA and vice versa. All, whole bacterial communities; Common: common bacterial communities; Rare: rare bacterial communities.

Neither deterministic (|betaNTI| > 2, 52.68%) nor stochastic processes (|betaNTI| < 2, 47.32%) dominated the assemblages of the whole bacterial communities (Figure 2A). However, the deterministic processes (variable selection) dominated the assemblages of common bacterial communities (84.64%, Figure 2A), while stochastic process dominated the assemblages of rare bacterial communities (25.70%, Figure 2A). In addition, the neutral assembly model confirmed that deterministic and stochastic processes were almost equal in all bacterial communities (Figure 2B). Deterministic and stochastic processes dominated the assemblages of common and rare taxa, respectively, verifying the results of betaNTI. Further, common taxa exhibited greater niche breadth values than rare taxa, and the species that occurred above and below predicted levels always had greater niche breadths when compared to those that occurred within the predicted range (Figure 2B).

We further determined the correlations between betaNTI and temporal distance, HA addition amount, and soil properties by partial Mantel tests (Figure 2C). The results showed that temporal distance always had greater effects than HA addition amount or soil properties. All soil properties except AN were significantly positively correlated with betaNTI, and the partial Mantel’s r was relatively lower between betaNTI and pH when compared to other factors. When compared to all and rare bacterial communities, the correlations between betaNTI of common taxa and temporal distance, HA addition amount, and most soil properties except pH and AN were much stronger.

We also employed an EAD method to determine the phylogenetic structure of a metacommunity to understand the microbial community assembly process by applying PD theory. The results showed that all 96 observed PDs were greater than the expected PDs, regardless of the number of species (Figure 2D), and the deviation between excepted PDs and observed PDs gradually decreased with the increased amount of HA added across all three datasets (Figure 2D and Supplementary Figure S4). Additionally, deviated PDs were always significantly positively correlated with species richness (Supplementary Table S8).

### Dynamics in Predicted Functions

**FIGURE 3:**
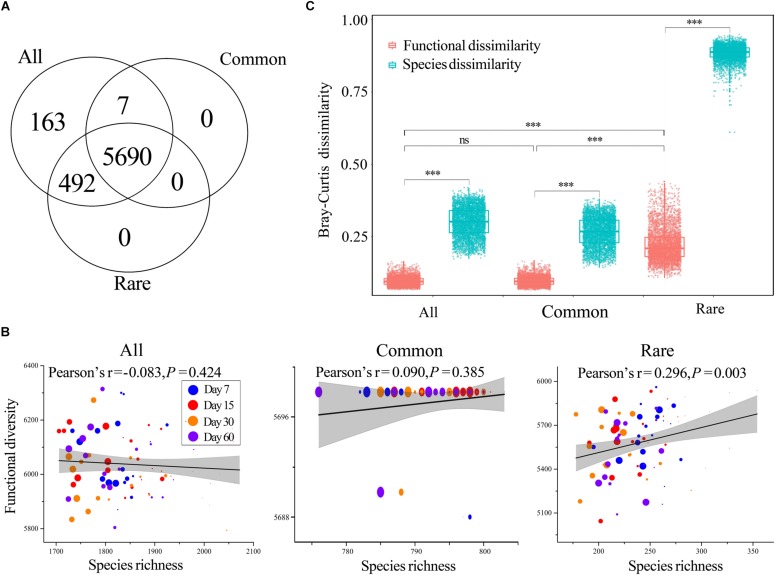
Functions of bacterial communities. **(A)** Number of shared KEGG orthologs (Kos) among all, common, and rare taxa. **(B)** Correlations between functional diversity and species richness of all, common, and rare taxa, respectively. The coefficients were determined by Pearson’s correlation tests. Circles of different color represent samples collected from different stages. Larger sizes indicate more added HA and vice versa. **(C)** Mean pairwise Bray–Curtis dissimilarity among functions. The top and bottom boundaries of each box indicate the 75^th^ and 25^th^ quartile values, respectively, and lines within each box represent the median values (*n* = 4560). ns, non-significant; ^∗∗∗^, a significant difference at the *P* < 0.001 level according to non-parametric Mann–Whitney *U-*test. All, whole bacterial communities; Common, common bacterial communities; Rare, rare bacterial communities.

The Tax4Fun yielded 6352, 5697, and 6182 KEGG orthologs (Kos) for all, common, and rare taxa, respectively. Venn analysis showed that almost all Kos of common taxa were a subset of that of rare taxa, whereas rare taxa had more unique Kos (Figure 3A) such as K00223 [EC: 1.3.1.71] (Delta24 (24(1))-sterol reductase), K02786 [EC: 2.7.1.69] (PTS system, lactose-specific IIA component), and K10551 [EC: 3.6.3.17] (D-allose transport system ATP-binding protein). Each Ko was treated as a functional trait, and we found that predicted functional diversity showed no significant difference among treatments across all three datasets (Supplementary Figure S5). In addition, two-way ANOVA also demonstrated that neither incubation time nor HA addition amount had significant effects on predicted functional diversity (Supplementary Table S9). The correlations between species richness and predicted functional diversity were tested by Pearson’s correlation test, and the results showed that predicted functional diversity was significantly positively correlated with species richness of rare taxa rather than all or common taxa (Figure 3B).

The Bray–Curtis dissimilarity of predicted functional composition showed no significant difference between all and common taxa, while the dissimilarity among rare taxa was much higher than that of all and rare taxa (Figure 3C). In addition, the predicted functional Bray–Curtis dissimilarity was always much lower than that of the Bray–Curtis dissimilarity for corresponding species, regardless of the number of taxa (Figure 3C).

### Factors Driving Structural and Predicted Functional Successions

**FIGURE 4:**
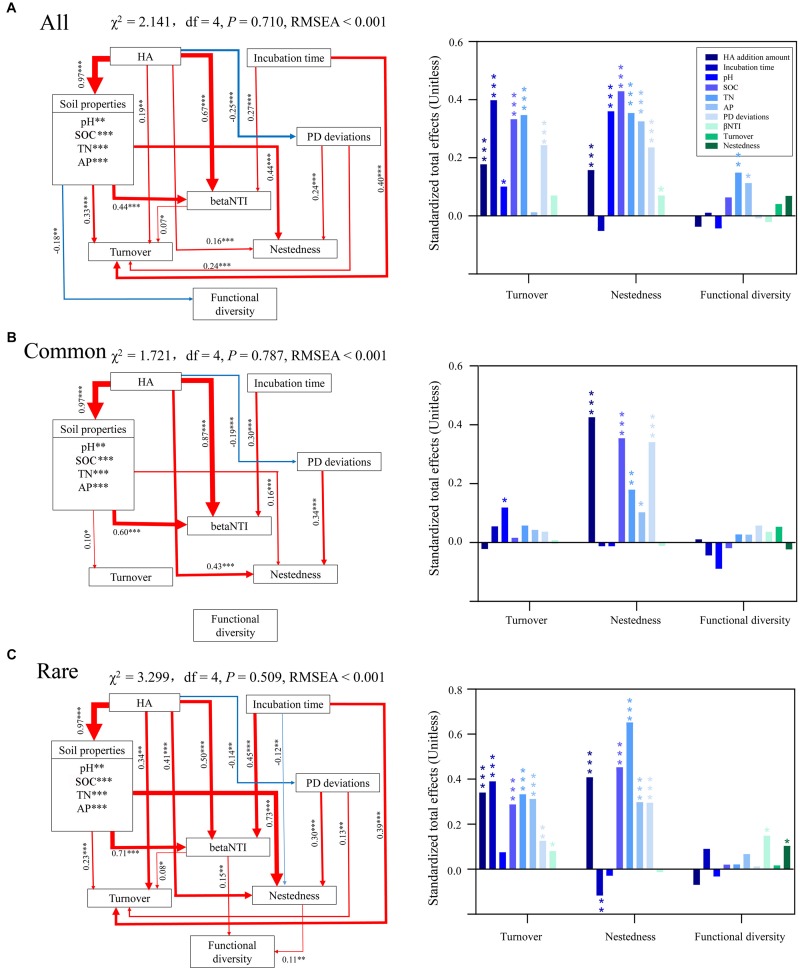
Direct and indirect effects of incubation time, HA addition amount, soil properties, species composition, and ecological processes on turnover, nestedness, and functional diversity of **(A)** all, **(B)** common, and **(C)** rare bacterial communities using structural equation modeling (SEM). Red lines indicate positive effects, while blue lines indicate negative effects. The width of arrows indicates the strength of significant standardized path coefficients (*P* < 0.05). Paths with non-significant coefficients are not presented. The right panel shows the corresponding standardized total effects of variations on turnover, nestedness, and functional diversity. ^∗^, ^∗∗^, and ^∗∗∗^ indicate significant effects at *P* < 0.05, 0.01, and 0.001, respectively. All, whole bacterial communities; Common, common bacterial communities; Rare, rare bacterial communities.

Random forest modeling was performed to determine the potential important factors influencing turnover, nestedness, and predicted functional diversity. Overall, incubation time had the greatest potential effect on turnover of all and rare taxa, while AP had the greatest potential effect on turnover of common taxa (Supplementary Table S10). SOC had the greatest potential effects on nestedness of common and rare taxa, while PD deviations were the most important potential factor affecting nestedness of all taxa. AP and nestedness had the greatest potential effect on predicted functional diversity of common taxa, while no factors had significant effects on predicted functional diversity of common taxa. Specifically, AN exerted no significant impact on predicted functional diversity of any bacterial communities. According to random forest modeling, we predicted the direct and indirect effects of incubation time and HA addition amount on turnover, nestedness, and predicted functional diversity using SEM (Supplementary Figure S6). The results showed that incubation time had the greatest effect on turnover of all and rare taxa, while pH was the most important potential factor affecting turnover of common taxa (Figure 4). SOC, HA addition amount, and TN had the greatest effects on nestedness of all, common, and rare species, respectively. TN and betaNTI had the greatest potential effects on predicted functional diversity of all and rare taxa, respectively. No factors showed significant effects on predicted functional diversity of common taxa, which was consistent with the results of random forest modeling (Supplementary Table S10) and the Pearson’s correlation test between predicted functional diversity and species richness (Figure 3B).

## Discussion

### Structural Successions of Common and Rare Taxa Under HA Amendment

Our results revealed distinct successional patterns in the community composition of common and rare bacteria under HA amendment. *Chloroflexi* (28.40%) and *Acidobacteria* (20.62%) dominated the whole bacterial communities (all taxa) and common bacterial communities (common taxa) (Supplementary Figure S1), suggesting the prevalence of oligotrophic taxa. In accordance with some previous studies on red soils (Li et al., 2017; Lynn et al., 2017), it may be because of the low pH and nutrient content in our testing soil (Supplementary Table S2).

Species richness decreased significantly with HA added amount (Table 2), and this should be because of the higher proportion of oligotrophic taxa (Supplementary Table S3), which negatively respond to nutrient amendment (Supplementary Table S4). Overall, common and rare taxa showed similar patterns in species richness, while rare taxa showed higher turnover than common taxa (Figure 1B and Supplementary Figure S2). Species richness was always correlated with environmental factors such as HA addition amount, SOC, TN, and AP, rather than incubation time (Table 2). The absolute values of slopes between environmental factors and species of rare taxa were always much higher than those of common taxa (Table 2). These findings suggested that the rare taxa were more sensitive to soil environmental changes in species richness (Cao et al., 1998). It should also be noted that species richness always peaked in treatment H1, suggesting that minor HA addition (5‰) can significantly promote species richness. This may be because HA provided nutrients such as SOC (1.36 g kg^–1^ higher than CK) and nitrogen (39.13 mg kg^–1^ higher than CK) (Supplementary Table S2) to bacteria, and the bacteria were resistant or resilient to such amendment (Allison and Martiny, 2008).

Both pairwise Bray–Curtis dissimilarity and sørensen’s index among all taxa were significantly greater than that among common taxa, but significantly lower than that among rare taxa (Figure 1A), suggesting that rare taxa contributed more to the discrimination of samples (Youssef et al., 2009; Galand et al., 2018). In addition to the Bray–Curtis dissimilarity, the turnover and the corresponding proportion of rare taxa were also much higher than those of common taxa (Figure 1B), and the common species exhibited greater niche breadth values than rare taxa (Figure 2B). Furthermore, turnover of rare taxa was more strongly correlated with both time-lag and difference in HA addition amount (Table 2). These results illustrated that the stable predominance of a few highly common species is contrasted by a highly dynamic turnover of rare species, as previously observed in marine bacterial communities (Gobet et al., 2012). The higher turnover of rare taxa could be due to that the rare species respond more quickly to environmental changes (Rosenzweig et al., 1994). As nestedness was much lower, we expected turnover to predominate but questioned if nestedness also was involved. Partial Mantel tests showed that there were significant correlations between differences in HA addition amount and nestedness in all, common, and rare bacterial communities (partial Mantel *P* < 0.001, Table 2), suggesting that nestedness was also involved in species succession. The nestedness could have resulted from several mechanisms, such as ordered extinctions (Sonnenburg et al., 2016) and differential dispersal abilities (Lomolino, 1996). The co-occurrence of these two divergent patterns of change in community composition suggested that there would also be more than a single ecological process underlying succession in the bacterial communities (Gao et al., 2019).

### Mechanisms Driving Successions in the Common and Rare Bacterial Communities

Determining the assembly of complex ecological communities is essential to revealing the mechanisms driving successions (Pagaling et al., 2014). Overall, our study demonstrated that the assemblages of common and rare taxa were distinct and were driven by divergent factors. Briefly, deterministic and stochastic processes dominated the assemblages of common and rare taxa, respectively (Figures 2A,B). We initially expected that the significant decrease in species richness (Supplementary Figure S2) and the remarkable discrimination in beta-diversity (Figure 1C) of all species would be a result of deterministic assemblage processes. However, the ecological stimulation model (βNTI) and neutral assembly model demonstrated that neither deterministic nor stochastic processes dominated the assemblages of the whole bacterial communities (Figures 2A,B). Instead, common taxa were mainly driven by deterministic assemblages (variable selection), while rare taxa were mainly driven by stochastic assemblages (Figures 2A,B), suggesting that the changes in alpha-diversity and beta-diversity of common and rare taxa may be because of different, but not mutually exclusive, assembly processes. Common taxa can occupy a wide variety of niches (Figure 2B) and competitively utilize an array of resources, supporting the notion that they may be strongly influenced by deterministic filtering (Umana et al., 2015). On the contrary, rare species always occupied narrow niche breadths, resulting in their being more strongly influenced by stochasticity (Orrock and Watling, 2010). In fact, regardless of whether the species were common or rare, our study found that species with wider niche breadths, which were mainly oligotrophic taxa (Supplementary Table S11), were always more strongly influenced by deterministic selection (Figure 2B). In addition, correlations between betaNTI and most environmental factors (HA addition amount and soil properties other than pH) of common taxa were always higher than those of rare taxa (Figure 2C), implying that the common species were more strongly influenced by environmental filtering.

Typically, organisms characterized by wider habitat niche breadths also exhibit higher environmental tolerances (Farjalla et al., 2012). Thus, although common taxa suffered strong environmental filtering (Dini-Andreote et al., 2015), the high environmental tolerances of community structure only caused slight variations in species richness and beta-diversity (Jiao et al., 2017a). Rare taxa also encountered environmental filtering, yet their high sensitivity to environmental disturbance could result in a remarkable change in community structure (Clarke and Murphy, 2006). In addition to environmental filtering, rare taxa are very vulnerable to ecological drift (Nemergut et al., 2013), which would contribute to stochasticity (Saleem, 2015) since slight negative changes in their abundance could result in their extinction (Pedros-Alio, 2006). Rare species are more likely to be influenced by ecological drift because of their low abundance and higher proportion of turnover partitioning in beta-diversity (Xue et al., 2018). Specifically, when selection is weak and species richness is low, ecological drift is the most important determinant driving the ecological assemblages of bacterial communities (Chase and Myers, 2011). These conditions were met in our study (Figures 2A,B and Supplementary Figure S2), as well as in some previous studies of wastewater treatment facilities (Ofiteru et al., 2010; Yang et al., 2011) and host-associated environments (Lankau et al., 2012).

The EAD method indicated that all observed PDs were greater than those of the expected PDs (Figure 2D), illustrating that the bacterial communities were affected by competitive exclusion, regardless of the number of species (O’Dwyer et al., 2012). It is worth noting that competitive exclusion can be either deterministic or stochastic (Segre et al., 2014). According to the results of the ecological stimulation model (βNTI) and neutral assembly model, we assumed that the competitive exclusion in common taxa may be deterministic, while it was stochastic in rare taxa. The deviation between excepted PDs and observed PDs gradually decreased as the amount of added HA increased (Supplementary Figure S4), implying a decrease in interspecific competition. This was not surprising since HA is rich in carbon and nitrogen (Wei et al., 2018), which makes it a good resource for bacteria, and greater resource availability is thought to reduce competition in microbial communities (Hubbell and Borda-De-Agua, 2004; Costello et al., 2012). Our study showed that species richness decreased as competition was reduced (Supplementary Table S8). Competition has been shown to play an important role in maintaining genetic diversity and driving speciation (Burger and Gimelfarb, 2004); thus, reduced competition may reduce speciation, resulting in a decrease in species richness.

### Predicted Functional Successions of Common and Rare Taxa Under HA Amendment

Most studies of the soil microbiome have emphasized the diversity associated with microbiomes rather than functions (Levy et al., 2018). In the present study, Tax4Fun was applied to investigate the potential functions of bacterial communities, and we also observed distinct predicted functional successions of common and rare taxa. Tax4Fun is limited in that only predicted functions can be analyzed; however, it provided useful information, such as comparative functional diversities of common and rare species, for our research. Our results showed that almost all Kos of common taxa were a subset of that of rare taxa, whereas rare taxa had more unique functions (Figure 3A), suggesting that rare taxa played an important role in maintaining the functional diversity of total bacterial communities. In addition, these unique functions contribute to the functional redundancy of the community (i.e., the insurance hypothesis) and enable the ecosystem to respond to and counteract environmental changes (Yachi and Loreau, 1999). The rare biosphere establishes a functional cache or resource pool for responding to disturbance events and harbors a persistent functional pool of ecological potential, which, through recruitment, may be broadly useful in promoting ecological stability (Lynch and Neufeld, 2015). For instance, in oil-contaminated soils, rare bacteria contribute a substantial fraction of auxiliary functions, such as carbohydrate-active enzymes, fermentation, and homoacetogenesis, which indicates their roles as sources of functional diversity (Jiao et al., 2017b) and verifies that rare microorganisms can mediate ecosystem function and stability.

Consistent with species composition, predicted functional dissimilarity among rare taxa was also significantly higher than that of all and common taxa, while common taxa were highly similar with whole communities in terms of predicted functional profiles (Figure 3C). These findings suggested that rare species are likely to be more functionally dissimilar from common ones and therefore expected to offer complementary or unique metabolic pathways to support community function (Dimitriu et al., 2010). Predicted functional dissimilarity was always much lower than species dissimilarity (Figure 3C), suggesting a higher stability of predicted functional composition when compared to species composition. We think this was clear evidence of functional redundancy in bacterial communities. Distinct functions can be taken over by different taxa or taxonomic groups, providing the ecosystem with a certain flexibility in the case of environmental events (Grossmann et al., 2016). That is, functional redundancy may work as a buffer, preventing ecosystems from losing essential functionalities when the species changes along with disturbance. In fact, a similar phenomenon has also been observed in a lake system, which demonstrates that ecosystem functions are often surprisingly stable despite considerable species turnover (Grossmann et al., 2016).

It is commonly believed that biodiversity loss can affect ecosystem functions and services (Saleem et al., 2019). However, although there was significant biodiversity loss as the amount of HA added increased in our study, predicted functional diversity only showed a significant correlation with species richness in rare taxa (Figures 3B, 4). We assume that the non-significant correlation between predicted functional diversity and species richness of all taxa could be because of the high quantity of species; thus, functions can be taken over by many potential species. The common bacterial communities with low quantities of taxa also showed such non-significant correlation, and we think this was because of the very low turnover and nestedness in common taxa (Figure 1B). Low turnover represents low species replacement and low nestedness represents less species extinction or speciation (Baselga, 2010). In other words, the species composition was more stable because of low turnover and nestedness, and the functions can thus remain stable. Correlations between predicted functional diversity and species richness of rare taxa showed a different pattern from those of all or common taxa. We postulate that this was a result of high turnover and nestedness in rare taxa (Figure 1B). Thus, we further deduce that there should be a turnover or nestedness threshold that can cause a significant change in functional diversity, and this threshold should be closely related to the size of the species pool.

## Conclusion

In the present study, we observed distinct successions in common and rare bacteria in soil under HA amendment. Common and rare taxa showed similar patterns in species richness, while rare taxa showed higher turnover than common taxa. The higher dissimilarity in rare species may have been a result of high species turnover and nestedness. Common species with wider niche breadths were found to be more strongly influenced by deterministic filtering than rare taxa, which occupied narrow niches and were primarily controlled by stochastic processes such as ecological drift. Generally, species with wider niche breadths were always more strongly influenced by deterministic selection. The analysis of predicted functions revealed that rare taxa occupied more unique functional traits than common taxa. Nestedness and betaNTI had direct effects on predicted functional diversity of rare taxa, while no factors showed significant effects on predicted functional diversity of common taxa. In addition, there was a significant positive correlation between species richness and predicted functional diversity in rare taxa, but not in common taxa. Overall, our findings highlight the structural and predicted functional successions of common and rare bacteria in soil under HA amendment, and provide a better understanding of the different functional responses of common and rare bacteria in soil to HA amendment.

## Data Availability Statment

The 16S rRNA gene sequences were submitted to the NCBI Sequence Read Archive (SRA) under the accession number SRP 169560.

## Author Contributions

All authors contributed intellectual input and assistance to this study and the manuscript preparation. ZL and PL developed the original framework. PL, JL, CJ, MW, and ML performed the experiments. PL did the data analysis and wrote the manuscript.

## Conflict of Interest

The authors declare that the research was conducted in the absence of any commercial or financial relationships that could be construed as a potential conflict of interest.

## Footnotes

1 http://qiime.org/

2 http://tax4fun.gobics.de/
