# Supplementary material for: Distinct Successions of Common and Rare Bacteria in Soil Under Humic Acid Amendment – A Microcosm Study
Source: Front Microbiol. 2019 Oct 1;10:2271. doi: 10.3389/fmicb.2019.02271 (PMC6779779; doi:10.3389/fmicb.2019.02271)
Supplement: Supplementary file 1 [file Data_Sheet_1.docx]

Supplementary Material

# Supplementary Figures and Tables

## Supplementary Figures

**Supplementary Figure S1** Species structure of all, common, and rare bacterial communities. All, whole bacterial communities; Common, common bacterial communities; Rare, rare bacterial communities.

**
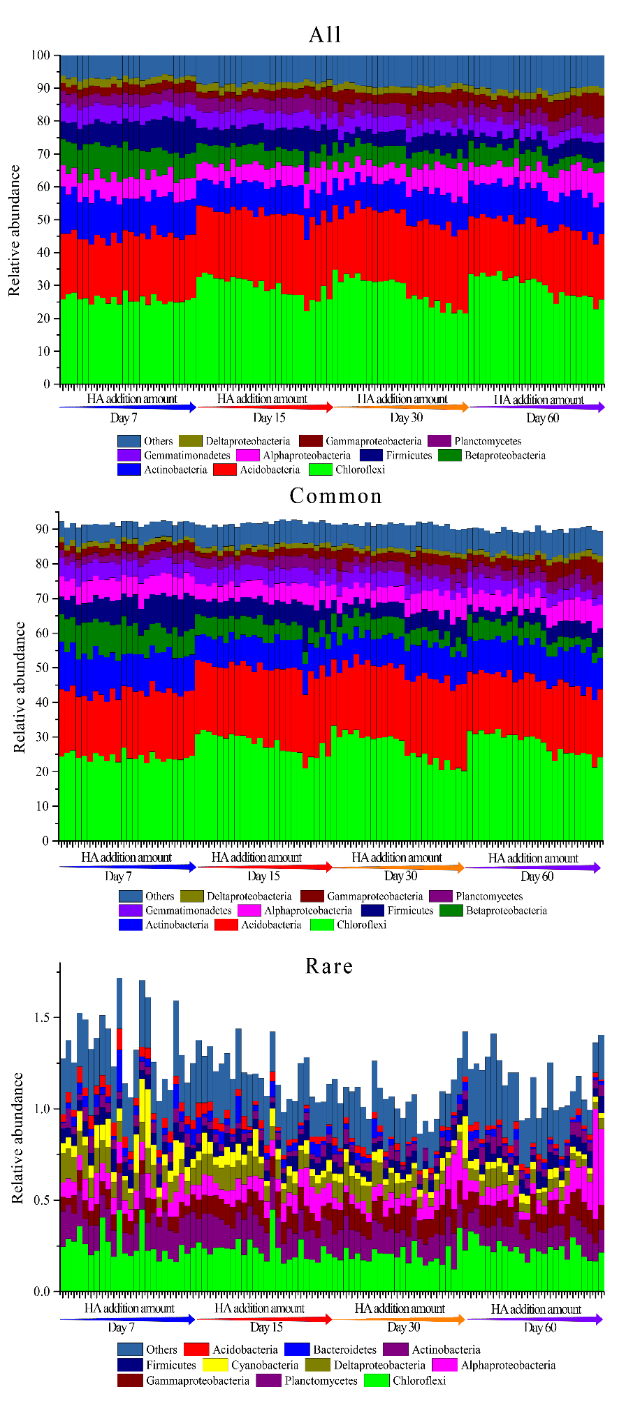
**

**Supplementary Figure S2** Boxplots showing the alpha-diversity indexes of each treatment. Different letters above bars indicate significant differences at the P < 0.05 level according to Duncan’s test. All, whole bacterial communities; Common: common bacterial communities; Rare: rare bacterial communities.

**
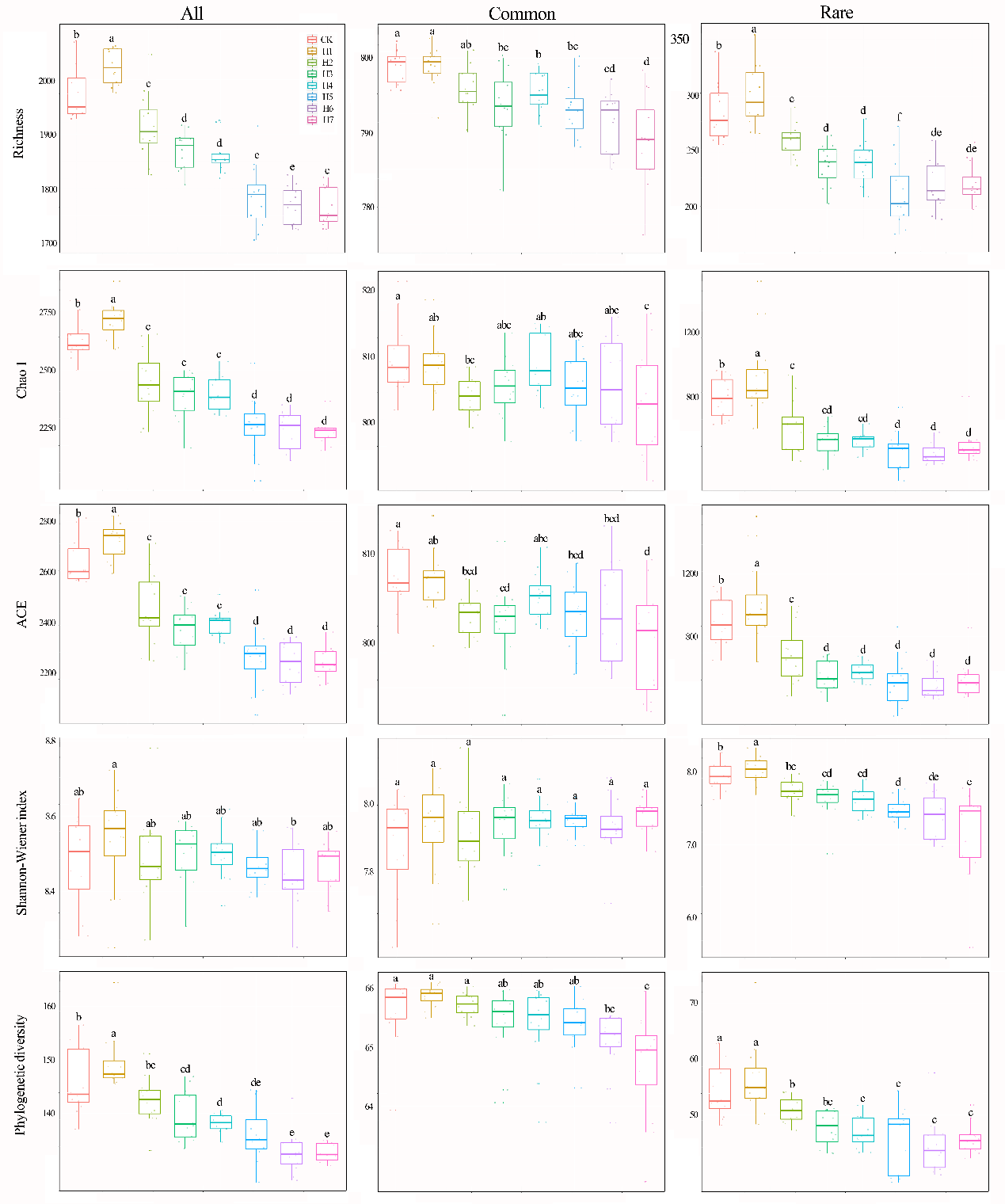
**

**Supplementary Figure S3** Correlations between species composition and incubation time, humic acid (HA) addition amount, and soil properties. Coefficients were determined by partial Mantel tests. Solid symbol represented non-significant correlation. TD, temporal distance. HA, difference in HA addition amount. All, whole taxa (n = 5153); Common, common taxa (n = 808); Rare, rare taxa (n = 2832).

**
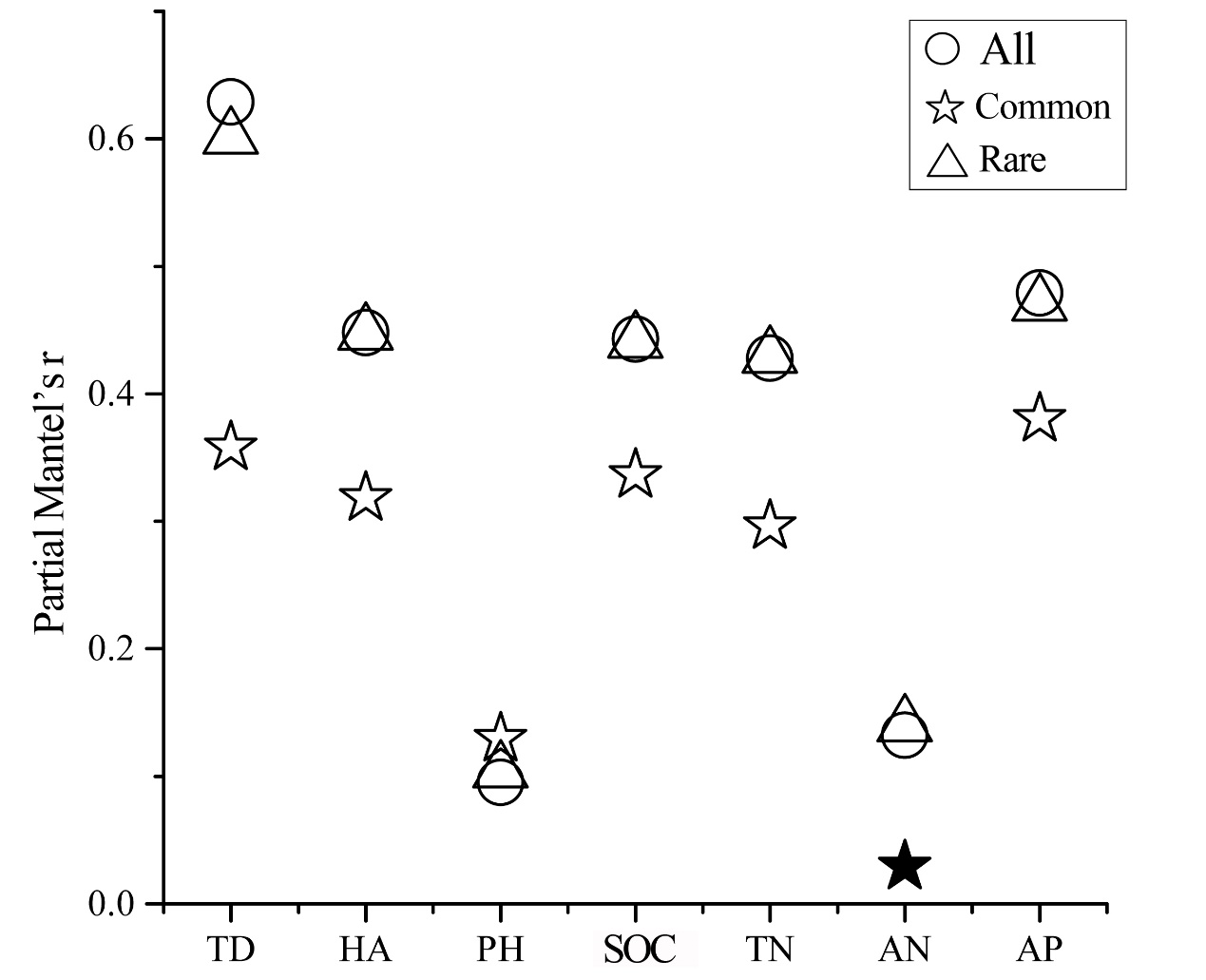
**

**Supplementary Figure S4** Correlations between humic acid (HA) addition amount and deviations between expected PDs and observed PDs. The coefficients were determined by Pearson’s correlation tests and Spearman’s correlation tests. All, whole bacterial communities; Common, common bacterial communities; Rare, rare bacterial communities.


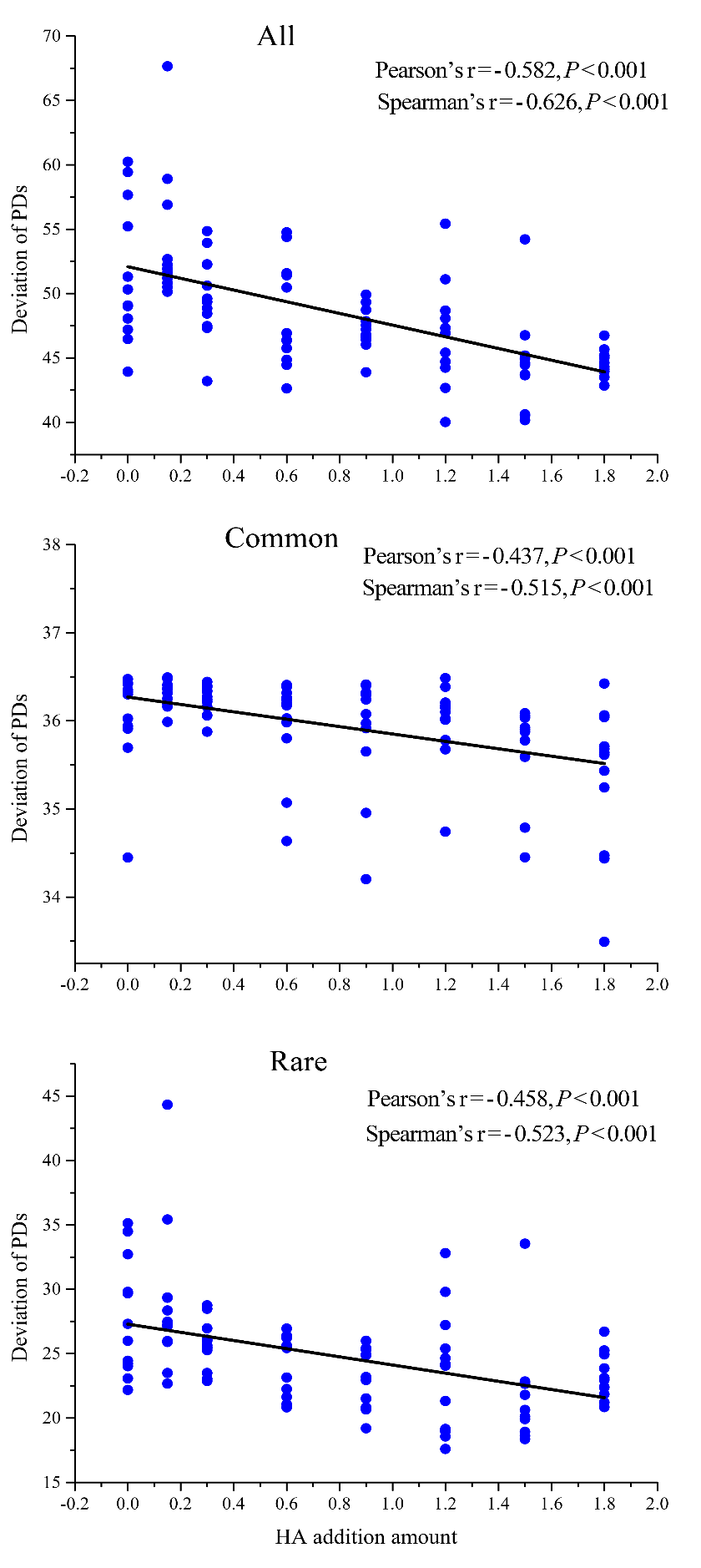


**Supplementary Figure S5** Functional diversity of bacterial communities. All, whole bacterial communities; Common, common bacterial communities; Rare, rare bacterial communities.

**
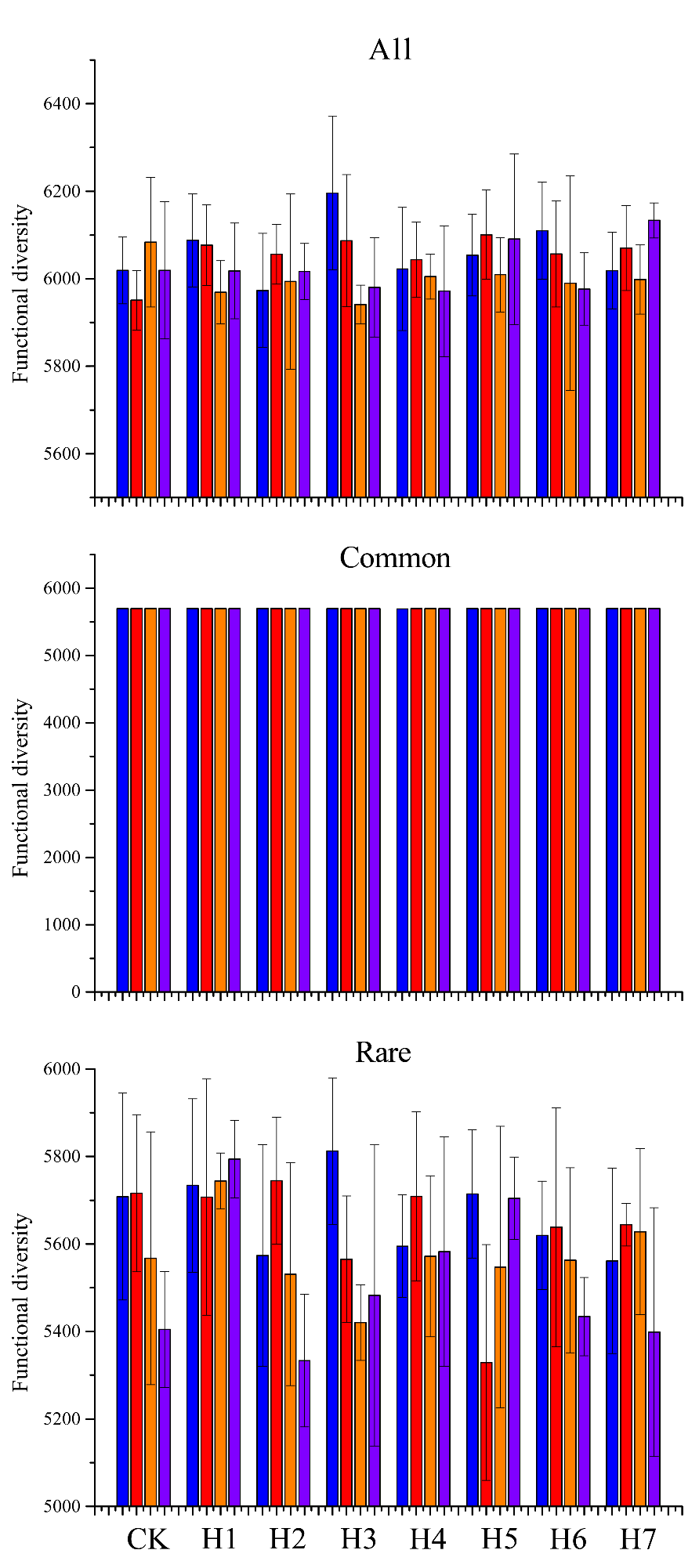
**

**Supplementary Figure S6** Prior model of structural equation modelling (SEM).


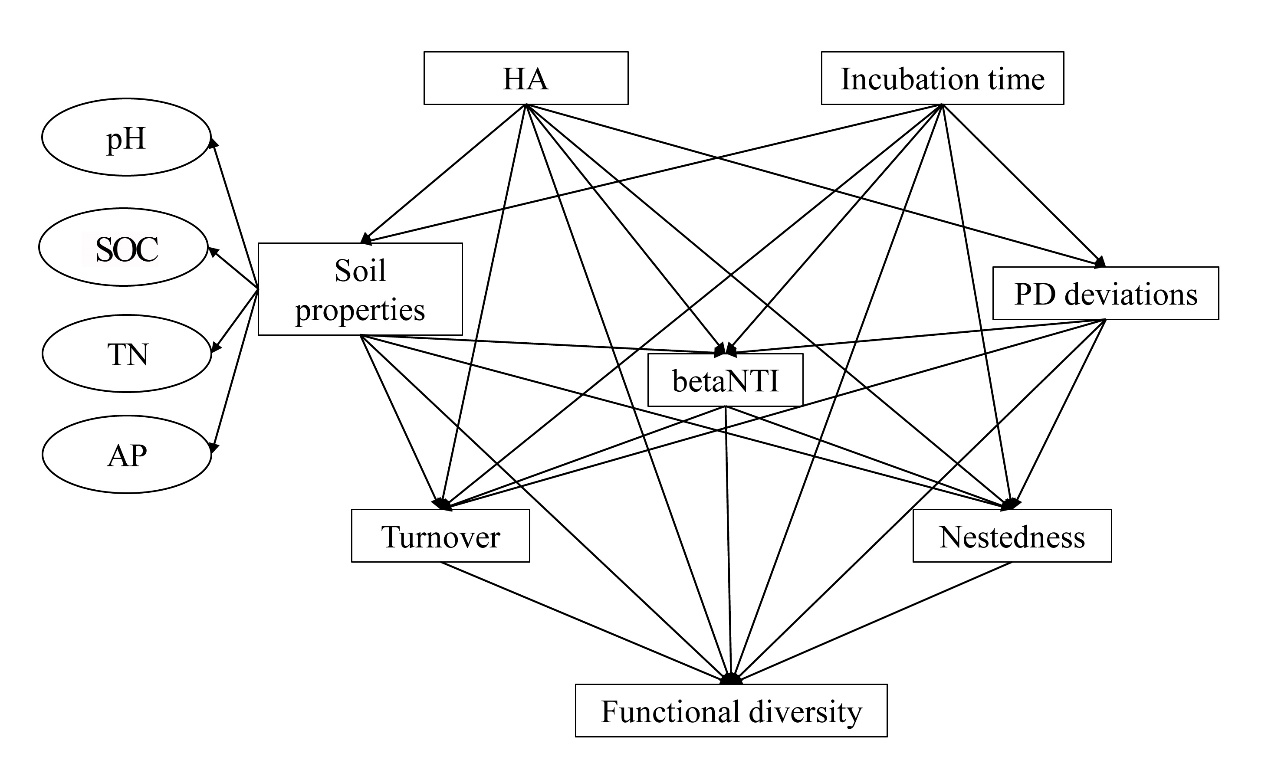


## Supplementary Tables

**Supplementary Table S1** The structural features of humic acid.

| **Elements** | **Proportion (%)** |
| --- | --- |
| C | 52.60 |
| O | 34.03 |
| H | 3.86 |
| N | 0.83 |
| S | 0.70 |
| P | 0.01 |
| **Structure** | **Proportion (%)** |
| aromatic C | 53.72 |
| alkyl C | 12.95 |
| Carbonyl C | 8.59 |
| aromatic C-O | 7.56 |
| O-C-O anomeric C | 7.31 |
| O-alkyl C | 6.92 |

**Supplementary Table S2** Soil physicochemical properties of each treatment.

|  |  | Day 7 | Day 15 | Day 30 | Day 60 |
| --- | --- | --- | --- | --- | --- |
| pH | | | | | |
|  | CK | (5.04±0.02) a | (4.75±0.03) c | (4.81±0.04) a | (4.81±0.03) a |
|  | H1 | (4.89±0.01) b | (4.80±0.04) b | (4.83±0.02) a | (4.90±0.04) a |
|  | H2 | (4.83±0.04) c | (4.89±0.01) a | (4.83±0.03) a | (4.91±0.02) a |
|  | H3 | (4.68±0.01) e | (4.89±0.02) a | (4.64±0.01) b | (4.80±0.07) a |
|  | H4 | (4.82±0.07) c | (4.87±0.02) a | (4.78±0.05) a | (4.82±0.05) a |
|  | H5 | (4.83±0.02) c | (4.64±0.02) d | (4.82±0.03) a | (4.83±0.05) a |
|  | H6 | (4.57±0.01) f | (4.43±0.06) e | (4.78±0.03) a | (4.68±0.01) b |
|  | H7 | (4.75±0.02) d | (4.75±0.02) c | (4.74±0.04) a | (4.86±0.04) a |
| SOC (g kg^-1^) | | | | | |
|  | CK | (1.72±0.57) h | (1.62±0.55) h | (1.47±0.59) h | (1.60±0.32) h |
|  | H1 | (2.85±0.64) g | (2.94±0.43) g | (3.10±0.45) g | (2.94±0.6) g |
|  | H2 | (4.68±0.60) f | (4.79±0.61) f | (4.47±0.74) f | (4.34±0.95) f |
|  | H3 | (7.78±1.76) e | (7.88±1.18) e | (7.30±1.18) e | (7.60±0.72) e |
|  | H4 | (20.18±0.63) d | (19.21±2.38) d | (20.2±0.64) d | (19.19±1.79) d |
|  | H5 | (23.50±2.45) c | (24.76±2.09) c | (24.63±0.93) c | (24.17±2.07) c |
|  | H6 | (29.42±1.26) b | (28.5±1.92) b | (28.87±1.94) b | (28.55±1.48) b |
|  | H7 | (34.15±2.38) a | (33.28±1.19) a | (34.12±1.95) a | (34.15±3.09) a |
| TN (mg kg^-1^) | | | | | |
|  | CK | (450.33±11.53) h | (456.00±7.65) h | (449.33±9.08) g | (460.13±17.01) h |
|  | H1 | (490.67±9.29) g | (472.67±12.50) g | (501.67±9.58) f | (508.33±8.03) g |
|  | H2 | (556.33±13.51) f | (541.10±13.01) f | (550.67±11.51) e | (556.33±15.03) f |
|  | H3 | (623.33±12.08) e | (651.67±13.06) e | (647.67±14.16) d | (662.67±11.85) e |
|  | H4 | (707.67±16.51) d | (693.33±15.51) d | (763.02±12.65) c | (751.33±11.58) d |
|  | H5 | (800.33±15.13) c | (795.33±11.53) c | (827.10±12.87) b | (817.67±19.61) c |
|  | H6 | (926.01±14.58) b | (866.67±13.51) b | (900.67±11.56) a | (902.33±14.16) b |
|  | H7 | (945.67±12.08) a | (932.67±23.53) a | (903.33±18.02) a | (972.20±21.63) a |
| AN (mg kg^-1^) | | | | | |
|  | CK | (33.67±2.52) c | (32.33±1.51) e | (41.01±2.14) a | (39.02±1.33) de |
|  | H1 | (45.67±3.06) ab | (36.33±1.49) d | (39.67±2.08) abc | (40.33±0.58) cd |
|  | H2 | (47.33±1.58) a | (37.67±1.53) cd | (40.22±1.21) ab | (47.27±2.21) a |
|  | H3 | (44.14±2.01) ab | (35.33±1.54) d | (39.01±2.11) abc | (43.06±1.07) b |
|  | H4 | (42.67±1.53) ab | (40.67±1.51) bc | (39.16±1.47) abc | (37.33±1.53) e |
|  | H5 | (45.67±1.17) ab | (40.33±2.31) bc | (36.67±2.51) c | (39.33±1.08) de |
|  | H6 | (44.33±1.15) ab | (45.13±1.01) a | (39.33±1.49) abc | (41.33±0.58) bc |
|  | H7 | (46.67±0.58) a | (43.02±2.00) ab | (37.67±0.58) bc | (45.67±1.43) a |
| AP (mg kg^-1^) | | | | | |
|  | CK | (19.6±0.12) f | (18.66±0.44) f | (19.98±0.36) e | (20.09±1.84) e |
|  | H1 | (21.30±1.21) e | (21.99±0.36) e | (21.18±0.75) d | (22.11±1.96) e |
|  | H2 | (21.85±0.97) e | (23.62±0.45) e | (23.45±1.51) d | (22.67±1.15) e |
|  | H3 | (25.42±0.11) d | (29.67±0.88) d | (28.63±0.99) c | (26.43±2.08) d |
|  | H4 | (33.31±2.13) c | (33.47±1.06) c | (33.51±1.38) b | (32.77±3.32) c |
|  | H5 | (31.11±1.21) c | (37.42±1.16) b | (44.46±2.28) a | (39.75±1.66) b |
|  | H6 | (39.39±0.09) b | (42.98±0.79) a | (47.98±3.13) a | (47.39±2.48) a |
|  | H7 | (42.50±1.29) a | (42.07±0.44) a | (48.87±2.66) a | (49.13±3.32) a |

**Supplementary Table S3** Relative abundance of oligotrophic and copiotrophic taxa in bacterial communities.

|  | All taxa | Common taxa | Rare taxa |
| --- | --- | --- | --- |
| oligotrophic | 54.30% a | 51.19% a | 0.43% b |
| copiotrophic | 34.37% b | 3.62% b | 0.49% a |

**Supplementary Table S4** Correlations between HA added amount and relative abundance of oligotrophic and copiotrophic taxa.

|  | All taxa | |  | Common taxa | |  | Rare taxa | |
| --- | --- | --- | --- | --- | --- | --- | --- | --- |
|  | r | *P* |  | r | *P* |  | r | *P* |
| oligotrophic | -0.288 | 0.004 |  | -0.258 | 0.011 |  | -0.268 | 0.008 |
| copiotrophic | 0.398 | < 0.001 |  | 0.351 | < 0.001 |  | 0.254 | 0.013 |

**Supplementary Table S5** Two-way ANOVA showing the effects of incubation time and humic acid (HA) addition amount on the species richness and other alpha-diversity indexes of bacterial communities.

|  |  | All taxa | |  | Common taxa | |  | Rare taxa | |
| --- | --- | --- | --- | --- | --- | --- | --- | --- | --- |
|  |  | *F* | *P* |  | *F* | *P* |  | *F* | *P* |
| Richness | | | | | | | | | |
|  | Time | 1.003 | 0.397 |  | 1.705 | 0.366 |  | 13.830 | < 0.001 |
|  | HA | **76.830** | **< 0.001** |  | **9.823** | **< 0.001** |  | **38.880** | **< 0.001** |
|  | Time×HA | 2.484 | 0.003 |  | 1.063 | 0.409 |  | 1.681 | 0.058 |
| Chao 1 | | | | | | | | | |
|  | Time | 2.686 | 0.054 |  | 1.428 | 0.243 |  | 4.892 | 0.004 |
|  | HA | **56.330** | **< 0.001** |  | **2.275** | **0.039** |  | **34.040** | **< 0.001** |
|  | Time×HA | 2.496 | 0.003 |  | 0.593 | 0.908 |  | 3.777 | < 0.001 |
| ACE | | | | | | | | | |
|  | Time | 1.819 | 0.153 |  | 0.221 | 0.882 |  | 3.580 | 0.019 |
|  | HA | **61.450** | **< 0.001** |  | **3.370** | **0.004** |  | **13.420** | **< 0.001** |
|  | Time×HA | 2.683 | 0.001 |  | 0.606 | 0.899 |  | 1.098 | 0.373 |
| Shannon-Wiener index | | | | | | | | | |
|  | Time | **31.530** | **< 0.001** |  | **31.990** | **< 0.001** |  | 4.572 | 0.006 |
|  | HA | 2.327 | 0.035 |  | 2.548 | 0.022 |  | **16.320** | **< 0.001** |
|  | Time×HA | 1.970 | 0.020 |  | 3.100 | < 0.001 |  | 2.394 | 0.004 |
| Phylogenetic diversity | | | | | | | | | |
|  | Time | 4.100 | 0.010 |  | 1.252 | 0.298 |  | 4.861 | 0.004 |
|  | HA | **9.885** | **< 0.001** |  | **5.238** | **< 0.001** |  | **31.130** | **< 0.001** |
|  | Time×HA | 1.255 | 0.240 |  | 1.120 | 0.352 |  | 2.815 | 0.001 |

Coefficients were determined by two-way ANOVA. HA, humic acid addition amount; Time, incubation time.

**Supplementary Table S6** Correlations between other alpha-diversity indexes and humic acid (HA) addition amount, incubation time, and soil properties.

|  | All taxa | Common taxa | Rare taxa |
| --- | --- | --- | --- |
| Pearson’s correlation between **Chao 1** and | | | |
| HA | -0.788*** | -0.255* | -0.623*** |
| Time | 0.083 | -0.067 | 0.083 |
| pH | 0.420*** | 0.135 | 0.322*** |
| SOC | -0.759*** | -0.220* | -0.600*** |
| TN | -0.786*** | -0.233** | -0.636*** |
| AN | -0.300** | 0.003 | -0.196 |
| AP | -0.743*** | -0.263** | -0.588*** |
| Pearson’s correlation between **ACE** and | | | |
| HA | -0.787*** | -0.362*** | -0.633*** |
| Time | 0.071 | 0.016 | 0.114 |
| pH | 0.416*** | 0.162 | 0.333*** |
| SOC | -0.754*** | -0.321*** | -0.603*** |
| TN | -0.791*** | -0.346*** | -0.645*** |
| AN | -0.280** | -0.085 | -0.221* |
| AP | -0.742*** | -0.339*** | -0.590*** |
| Pearson’s correlation between **Shannon-Wiener index** and | | | |
| HA | -0.184 | 0.206* | -0.663*** |
| Time | 0.036 | -0.045 | -0.230** |
| pH | 0.344** | 0.171 | 0.250** |
| SOC | -0.189 | 0.198 | -0.642*** |
| TN | -0.173 | 0.206* | -0.650*** |
| AN | 0.038 | 0.085 | -0.265** |
| AP | -0.218* | 0.174 | -0.694*** |
| Pearson’s correlation between **phylogenetic diversity** and | | | |
| HA | -0.749*** | -0.492*** | -0.613*** |
| Time | 0.051 | 0.001 | -0.144 |
| pH | 0.334*** | 0.160 | 0.270** |
| SOC | -0.729*** | -0.486*** | -0.592*** |
| TN | -0.746*** | -0.475*** | -0.634*** |
| AN | -0.341*** | -0.237** | -0.224** |
| AP | -0.708*** | -0.502*** | -0.623*** |

Coefficients were determined by Pearson’s correlation test. HA, humic acid addition amount; Time, incubation time; SOC, soil organic carbon; TN, total nitrogen; AN, available nitrogen; AP, available phosphorus; *, **, and *** indicate significant correlations at *P* < 0.05, 0.01, and 0.001, respectively.

**Supplementary Table S7** Two-way PERMANOVA showing the effects of incubation time and humic acid (HA) addition amount on the species composition.

|  |  | All taxa | |  | Common taxa | | | |  | Rare taxa | | | |  |
| --- | --- | --- | --- | --- | --- | --- | --- | --- | --- | --- | --- | --- | --- | --- |
|  |  | *F* | *P* |  | *F* | | *P* | |  | *F* | | *P* | |  |
| Incubation time | | **30.546** | **< 0.001** | |  | **39.809** | | **< 0.001** | |  | **2.343** | | **< 0.001** | |
| HA addition amount | | 7.224 | < 0.001 | |  | 9.206 | | < 0.001 | |  | 1.453 | | < 0.001 | |
| Incubation time ×HA addition amount | | 1.780 | < 0.001 | |  | 1.872 | | < 0.001 | |  | 1.083 | | 0.002 | |

**Supplementary Table S8** Correlations between deviated PDs and species richness of all, common, and rare taxa.

|  | r | P |
| --- | --- | --- |
| All taxa | 0.658 | <0.001 |
| Common taxa | 0.548 | <0.001 |
| Rare taxa | 0.589 | <0.001 |

Coefficients were determined by Pearson’s correlation test.

**Supplementary Table S9** Two-way ANOVA showing the effects of incubation time and humic acid (HA) addition amount on the functional diversity.

|  |  | All taxa | |  | Common taxa | | | |  | Rare taxa | | | |  |
| --- | --- | --- | --- | --- | --- | --- | --- | --- | --- | --- | --- | --- | --- | --- |
|  |  | *F* | *P* |  | *F* | | *P* | |  | *F* | | *P* | |  |
| Incubation time | | 1.346 | 0.267 | |  | 0.667 | | 0.576 | |  | 2.480 | | 0.069 | |
| HA addition amount | | 0.354 | 0.925 | |  | 0.573 | | 0.775 | |  | 1.184 | | 0.325 | |
| Incubation time ×HA addition amount | | 0.709 | 0.808 | |  | 1.048 | | 0.424 | |  | 1.076 | | 0.396 | |

**Supplementary Table S10** Potential factors affecting functional diversity, turnover, and nestedness.

|  |  | HA | TIME | pH | SOC | TN | AN | AP | PD | betaNTI | Turnover | Nestedness | R^2^ | *P* |
| --- | --- | --- | --- | --- | --- | --- | --- | --- | --- | --- | --- | --- | --- | --- |
| To functional diversity | All taxa | 10.186** | 8.722** | 8.626** | 13.689** | 11.637 | 5.1 | **15.832**** | 13.058** | 5.145 | 10.697* | 14.155** | 0.157 | 0.01 |
|  | Common taxa | 4.9585 | 6.328 | 8.0735 | 8.476 | 7.611 | 3.6825 | 7.6795 | 5.1685 | 5.629 | 9.9465 | 8.2035 | 0.048 | 0.33 |
|  | Rare taxa | 10.813* | 11.053** | 6.195 | 11.598 | 12.420** | 1.425 | 10.813** | 2.019 | 8.401** | 5.803 | **11.268**** | 0.116 | 0.01 |
| To turnover | All taxa | 16.051** | **61.917**** | 34.044** | 23.134** | 19.648** | 21.141** | 26.310** | 31.733** | 21.439** | - | - | 0.673 | 0.01 |
|  | Common taxa | 15.909** | 17.557** | 24.317** | 25.864** | 29.498** | 17.599** | **27.651**** | 21.737** | 19.237** | - | - | 0.435 | 0.01 |
|  | Rare taxa | 18.455** | **48.294**** | 20.867** | 26.932** | 25.383** | 13.375** | 27.445** | 3.652 | 16.994** | - | - | 0.382 | 0.01 |
| To nestedness | All taxa | 18.805** | 25.933** | 29.014** | 21.147** | 17.271** | 16.921** | 23.701** | **29.451**** | 17.689** | - | - | 0.762 | 0.01 |
|  | Common taxa | 11.953** | 17.371** | 10.494** | **26.644**** | 20.078** | 15.489** | 23.083** | 20.833** | 10.712** | - | - | 0.355 | 0.01 |
|  | Rare taxa | 16.279** | 25.847** | 29.038** | **30.359**** | 15.279** | 25.538** | 20.113** | 6.830* | 19.804** | - | - | 0.584 | 0.01 |

The importance of each predictor was determined by assessing the decrease in prediction accuracy when the data for the predictor was randomly permuted. SOM, soil organic matter; AP, available phosphorus; TN, total nitrogen; AN, available nitrogen; PD deviation, deviation between excepted PD and observed PD; *, *P* < 0.05; **, *P* < 0.01.

**Supplementary Table S11** Proportion of oligotrophic and copiotrophic taxa that occurred out of neutral assembly model predictions.

|  | All taxa | Common taxa | Rare taxa |
| --- | --- | --- | --- |
| oligotrophic | 43.10 % | 49.07 % | 41.53 % |
| copiotrophic | 36.52 % | 36.93 % | 37.96 % |
